# A Novel Application of 3D Printing Technology Facilitating Shell Wound Healing of Freshwater Turtle

**DOI:** 10.3390/ani12080966

**Published:** 2022-04-08

**Authors:** Tsung-Fu Hung, Po-Jan Kuo, Fung-Shi Tsai, Pin-Huan Yu, Yu-Shin Nai

**Affiliations:** 1 WeCare Animal Medical Center, 1F., No. 270, Daxing Rd., Taoyuan Dist., Taoyuan City 334, Taiwan; 2 Tri-Service General Hospital, School of Dentistry, National Defense Medical Center, Taipei City 114, Taiwan; 3 Momonga Exotic Animal Hospital, No. 20, Section 4, Chongxin Rd., Sanchong District, New Taipei City 241, Taiwan; junior10873@msn.com; 4 Institute of Veterinary Clinical Science, National Taiwan University, Taipei City 106, Taiwan; pinhuan@ntu.edu.tw; 5 Department of Entomology, National Chung Hsing University, Taichung City 402, Taiwan

**Keywords:** 3D printing technology, freshwater turtle, *Ocadia sinensis*, shell wound healing

## Abstract

**Simple Summary:**

This report describes how to apply the combination of 3D scanning, computer-aided design (CAD), and 3D printing to make a protective device for rescuing wild animals. In recent years, although 3D tools have become relatively low-cost and reachable, veterinary medical applications based on this technology are quite limited. The present article successfully extricates a wild freshwater turtle from an extensive shell defect within a short period. Integration of multiple sciences to 3D technology can provide a facile model for veterinary medical applications.

**Abstract:**

Numerous cases and a shortage of resources usually limit wild animal rescue. New technology might save these severely injured wild animals from euthanasia by easing the requirement of intensive medication. Three-dimensional (3D) technologies provide precise and accurate results that improve the quality of medical applications. These 3D tools have become relatively low-cost and accessible in recent years. In the medical field of exotic animals, turtle shell defects are highly challenging because of inevitable water immersion. This report is the first attempt to apply the combination of 3D scanning, computer-aided design (CAD), and 3D printing to make a device that protects the wound from exposure to water or infection sources. The presented techniques successfully extricate a wild freshwater turtle from an extensive shell defect within a short period. Integration of multiple sciences to 3D technology can provide a facile model for veterinary medical applications.

## 1. Introduction

In exotic animal medicine, shell trauma is a frequent casualty for wildlife turtles and tortoises [1,2,3]. The shell fractures were usually restored by external fixation utilizing orthopaedic screws, orthopaedic pins, surgical wiring, epoxy, or super glue to bridge fracture segments [4,5,6,7]. These procedures provided instant protection and fixation of shell fracture [1,3]. However, a significant shell defect involving the shoulder, pelvic area, or penetrating punctures of the coelom could lead to a poor or even grave prognosis [3]. In the case of missing fragments, the shell usually cannot be stabilized and rehabilitated, which may be fatal without adequate medication and intensive nursing. Keeping the wound moist and clean is ideal for wound healing [1,3,7]. Although reptile wound healing is relatively slow compared with that of mammals, extensive shell loss becomes harsher and more challenging for freshwater turtles. The water turtle must maintain an everyday life for nutrition, urination, and defecation within the water environment [2]. Therefore, inevitable water immersion and faecal contamination may cause recurrent infections and delay wound healing. Thus, prolonged and intensive nursing becomes a medical burden [3].

Three-dimensional (3D) technology has been utilized in many medical applications thanks to its high precision and accuracy [8,9,10]. Recently, based on the development of 3D fabrication and medical imaging technology, increasingly affordable 3D printing technologies now make it possible to create highly customizable patient-tailored products [11].

Herein, we describe a feasible way to facilitate the recovery of a wild freshwater turtle from the delayed healing of severe and extensive shell defects, which reduces the intensity and frequency of medical care. The computer-aided design (CAD) and the fused deposition modelling (FDM) 3D printer could rapidly fabricate a customized device, which provided physical support of the defective shell structure. In addition, the device protected the delayed healing wound from water exposure. After the delivery of the device, the burden of intensive care was drastically decreased, and the wound infection eased rapidly, which favoured wound healing. We tracked the wound condition in the fifth month, and one year after the delivery of the device, the follow-up results showed a favourable prognosis and the subject was free from adoption without special care.

The purpose of this investigation was to develop novel attempts to apply 3D printing technology to wild freshwater turtles for wound healing; the technique provides an alternative option for exotic animal medicine.

## 2. Materials and Methods

### 2.1. Animal Background

**Figure 1:**
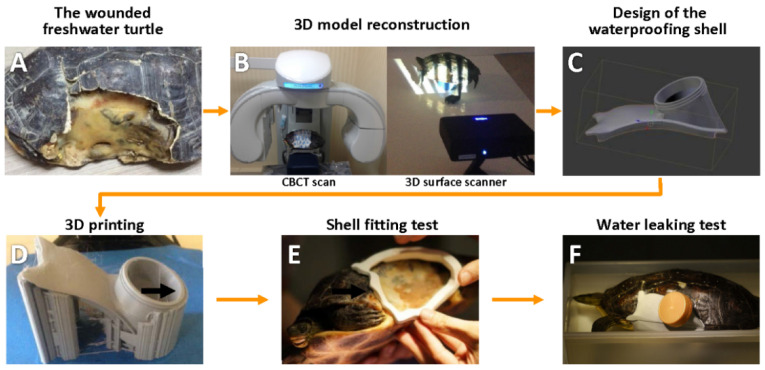
**Flowchart of the treatment plan.** (**A**) The female freshwater turtle (*Ocadia sinensis*) with a 13 cm long oval defect; (**B**) The wounded freshwater turtle is scanned by a CBCT and 3D surface scanner for reconstruction of the 3D model; (**C**) the reconstruction of a hard tissue 3D model and defect surface model are applied to design a waterproofing shell; (**D**) all 3D models of the waterproofing shell are used to fabricate the template by a fused deposition modelling (FDM) type Delta 3D print; (**E**) waterproofing shell fitting test is performed for secure fixation; (**F**) water leaking test.

A female freshwater turtle (*Ocadia sinensis*) was found and rescued by a local wildlife rescue association in Taiwan. This 30 cm long turtle had a severe shell defect on the left side (Figure 1A). The coelomic membrane was exposed on the fracture site, and debris, dirt, and fly larva were present around the affected area. After emergency wound management by a volunteer of the association, the turtle was transferred to the veterinary hospital. After two months of intensive care from a veterinarian, including systemic antibiotics, wet-to-dry docking [3], and supportive treatment with irrigation, flushing, and nutrition, the wound remained with minor infection, and healing was delayed.

### 2.2. Diagnosis and Treatment Plan

All procedures were reviewed and approved by the Bureau of Forestry, Council of Agriculture of Taiwan, the competent authority of wildlife utility (project 106 FD-08.3-C-28). In the veterinary hospital, the turtle presented with a 13 cm long oval shell lost on the left side involving the carapace, plastron, and most parts of the bridge (Figure 1A). The left anterior edge of the shell bridge was lost; thus, the support structure between the carapace and plastron was disconnected. A fistula wound with purulent discharge was found on the caudal side of the coelomic membrane, and several minor wounds spread around the membrane with purulent discharge. Systemic infection was found based on the increased quantity of white blood cells. Therefore, systemic antibiotic therapy was used (metronidazole 20 mg/kg b.i.d. with trimethoprim 30 mg/kg b.i.d. under subcutaneous injection [SC] for 14 days). The wound on the exposed choelomic membrane did not heal well, with recurrent infections even after 2 months of intensive care.

The daily care procedure included removing the dirty bandage, wound irrigation (sterile Ringer’s solution 10 mL/kg SC, s.i.d.), and nutrition support (oral gavaging the commercial critical care diet at a dose of 3% of body weight), which may require a team of at least two people to complete. Moreover, the dressing was not waterproof to keep the defect dry. Therefore, the wound was difficult to keep clean and reduce possible bacterial infections at the wound site for this freshwater turtle, especially when there was a shortage of human medical resources. The novel device as the customized shell was designed and fabricated following the flow chart (Figure 1) to provide mechanical support and water isolation. All device design and test steps were performed and completed under the supervision of a veterinarian. In addition, the pain and stress behaviour of the turtle was carefully monitored during treatment.

### 2.3. The Bone Model and Surface Model of Defect Reconstruction

The turtle underwent cone-beam computed tomography (CBCT) (Newtom 5G, Imola, Italy) scanning (Figure 1B left). The CBCT scan was obtained with a 0.3 mm voxel size, power of 110 kVp, 5 mA, 10 s exposure, and all raw data were compacted in DICOM format for further radiographic diagnosis. The skeletal 3D model was reconstructed after adjusting the density until the tissue border was clearly identified in the software (OsiriX MD, Pixmeo, Geneva, Switzerland). The 3D reconstructed model was exported as a stereolithography (STL) file for further editing. The surface model of the defect was acquired by a structured light 3D surface scanner (Einscan-S, Shining 3D, Hangzhou, China) (Figure 1B right) and exported as an STL file. All 3D models were edited by Blender, a computer-aided design software (Blender, Blender Foundation, Amsterdam, The Netherlands). The bone and surface models were carefully aligned and merged to show the locale of the bone edge under the scutum to confirm any bone degeneration in the case of underlying infection.

The bone model was mainly used for diagnosis and skeletal structure analysis, which indicated the mechanical force support area. Moreover, the surface model provides the detailed architecture and morphology of the defect and surrounding shell structure for the device outline design. The edited models were processed by slicer software (Kisslicer, Jonathan Dummer, Orlando, FL, USA; Simplify3D, Simplify3D Inc., Cincinnati, OH, USA) and printed by a fused deposition modelling (FDM)-type Delta 3D printer (Atom2, ALT, Taipei, Taiwan) with modified polylactic acid (PLA) filaments (Gypsum, Next Print, Tainan, Taiwan).

### 2.4. Design, Construction, and Fitting of the Protective Device

**Figure 2:**
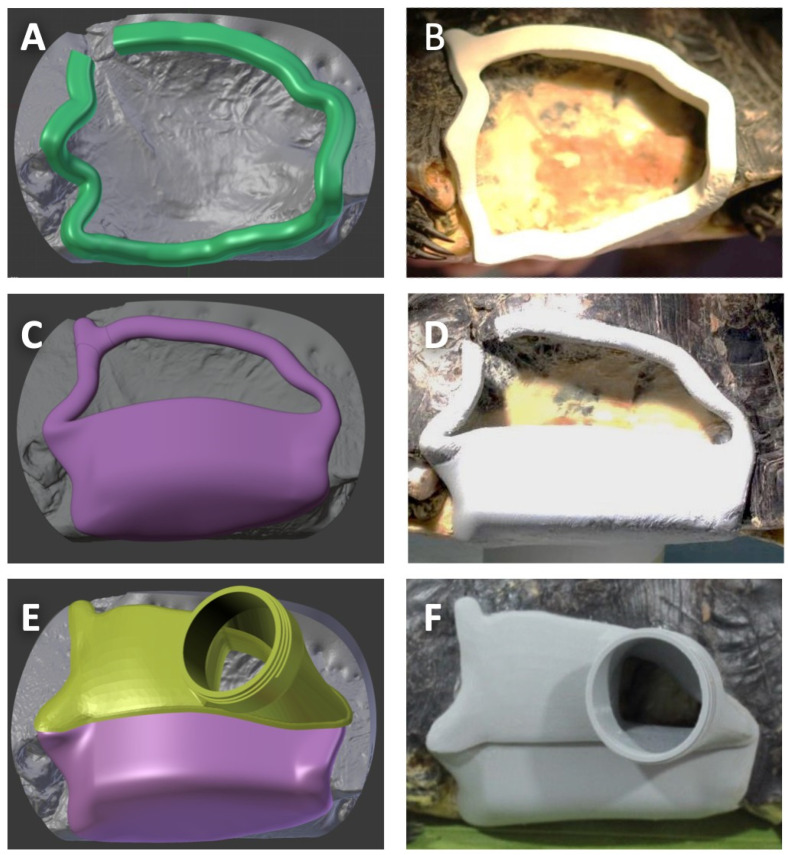
**Design and fitting of the protective device on the shell defect.** 3D reconstructed models of protective device: (**A**) the CAD model of snap-fit joints rim structure (green part); (**B**) printed and fitting tested on the defect; (**C**) a hull structure (purple part) was designed according to the previously designed rim structure; (**D**) printed and fitting tested again on the defect; (**E**) a model of the cover with a threaded porthole (yellow part) was designed; (**F**) printed and installed on the hull structure for further testing.

The shell defect formed several undercuts on the carapace, plastron, and caudal part of a bridge, which would facilitate fixation; however, the lost anterior edge of the skeletal bridge was a challenge for waterproofing owing to the unstable soft tissue during left forelimb movement. According to the reconstructed model, we designed a protective device (Figure 1C) and printed it (Figure 1D) as described previously. The device practicability was tested through fixation stability (Figure 1E), not interfering with animal activity, and waterproofing (Figure 1F). A rim was designed and fabricated with a snap-fit groove to tightly engage the overhanging shell’s undercuts (Figure 2A). The junction between the shell edge/soft tissue and the rim was diminished, and the groove of the rim was covered over the edge of the defect for at least 5 mm to achieve secure fixation. The prototype of the rim showed promising engagement with the printed defect model (Figure 2B). After fixation, the rim was further remodelled to form a hull shape, with an opening kept above (Figure 2C). The rim with a hull structure was printed and tested again on the defect for stability (Figure 2D). To reduce the chance of leakage, the junction between the rim groove and shell defect edge/soft tissue was filled with polydimethylsiloxane (PDMS) as a sealant (Sylgard 184, Dow Corning, Midland, MI, USA). A layer of silicone sealant (Silicone sealant, Dow Corning, Midland, MI, USA) was applied to the outer surface between the shell and rim. Furthermore, a cover part was designed with a porthole to attach to the opening (Figure 2E), and it was printed and secured on the opening of the hull structure (Figure 2F). The edge of the cover, which contacts a layer of silicone sealant, coated the opening to close the waterproof opening. The porthole was designed with thread to fit a wide-mouth bottle cap to facilitate daily care [12].

### 2.5. Daily Care and Wound Management

The hull and cover were fixed as designed and would not be removed during daily care. The porthole remained open during the dry docking and closed with the cap when the turtle was immersed in the water. The cap was removed approximately for 6~8 h daily to avoid high humidity that could induce fungal organism colonization. The caudal pocket wound of the defect was cleaned daily and debrided through the porthole. The device’s margin fitness was assessed once every month, and the sealant was added if a crack or leakage was noted.

## 3. Results

### 3.1. Device Fitting and Leakage Test

The delivery of the rim structure attested to the feasibility of noninvasive fixation. The rim was adapted to the defect and allowed the turtle to stay in a room for 3 h. The fitness of the rim was well adapted, and the shape of the rim with hull structure was modified to slim (Figure 2D,E), while the original design of the rim was too thick that it might scratch and be pushed by the turtle’s limbs. The hull structure was installed and rechecked as described above. The device attached to the defect was stable and was not reachable by animal activity. After delivery, animal behaviour presented no difference, including scratching, constant hiding, unwillingness to move, and activity change. Clean gauze was placed in the hull without the cover, and the patient was half-immersed and constrained in water. After 1 h, the gauze was dry, indicating no water leakage into the hull. Moreover, the total weight of the device was approximately 110 g, and it affected the physiological activities very little.

### 3.2. Patient Follow-Up

**Figure 3:**
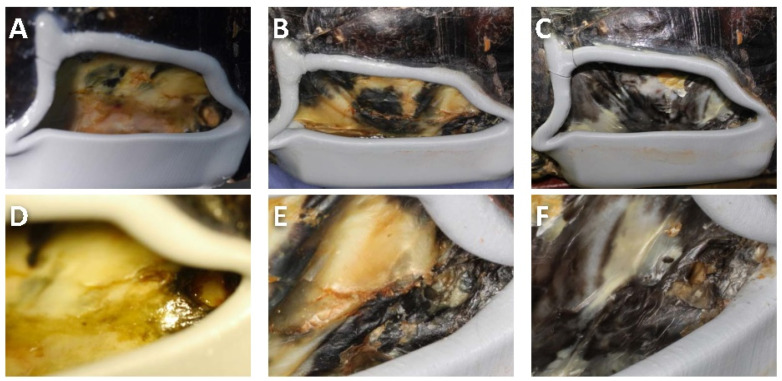
**Clinical photos after device application without cover structure:** (**A**–**C**) lateral view; (**D**–**F**) direct view of soft tissue damage at the defect posterior area; (**A**) fragile tissue at day 0; (**B**) small keratinized areas presentation at 3-month recall; (**C**) mature keratinized tissue formation at 5-month recall; (**D**) fragile tissue with exudate formation at day 0; (**E**) keratinized tissue formation at 3-month recall; (**F**) complete closure of fistula and mature keratinised tissue presentation at 5-month recall.

In the clinical observation, the affected area presented debris without applying the device to protect the defect. The exudate formation and chronic infection did not subside at this stage (Figure 3A). After fabricating and applying the protective device, the device could cover the defect and avoid wound contamination during the healing process. This device provided waterproofing and reduced the wound infection risk during immersion. The appetite and activity of the patient significantly increased one month after applying the device. In addition, under a veterinarian’s assessment of pain and distress, the patient did not show any pain-like (nocifensive) behaviour. Keratinization was present on the coelomic membrane (Figure 3B), and the counts of white blood cells were in the normal range (1.4 × 10^4^/μL) at the one-month recall after device application.

**Figure 4:**
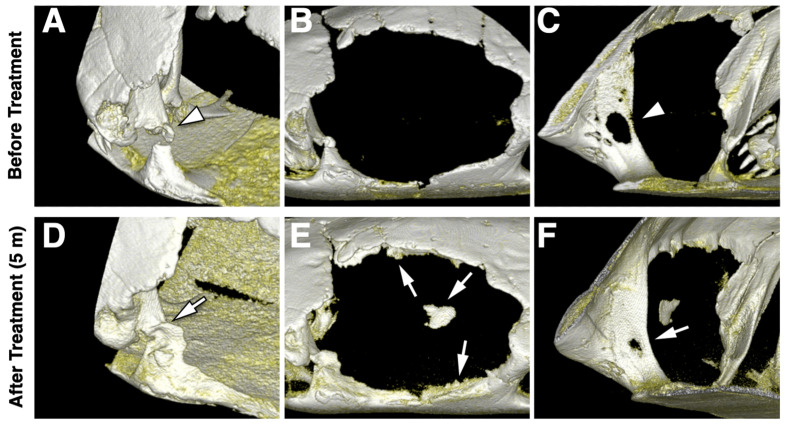
**CBCT three-dimensional images of initial condition (A–C) and 5 months after device application (D–F):** (**A**) hard tissue defect on the anterior border (arrowhead); (**B**) defect lateral view; (**C**) hard tissue fenestration on the posterior border (arrowhead); (**D**) fracture healing (arrow) on the anterior border; (**E**) new hard tissue formation (arrow) at defect border and centre area; (**F**) hard tissue healing (arrow) on the posterior border of the defect.

The matured keratinized tissue almost covered the defect surface at five months of reexamination, and the wound was solid during palpation (Figure 3C). The tissue damage on the posterior defect border healed without exudate discharge, and a leather-like granulation bed was in place (Figure 3D–F). Using the CBCT 3D model to assess hard tissue formation, bony bridge development was observed at the fracture site of the anterior area (Figure 4A,D). Ossification was present on the border and the central location of the defect. Moreover, the fenestration lesion on the posterior side was reduced (Figure 4C,F).

Through 12 months of follow-up, the device did not loosen and maintained stability. The turtle maintained normal physiologic functions, including swimming ability, buoyancy, and ambulation, during daily access to an underwater environment. No adverse effects inducing pain or stress were noted.

## 4. Discussion

One of the most common current issues encountered in wild freshwater turtles is shell injury. Most of these injuries are caused by road traffic accidents and predator attacks [3]. In the present report, we described the workflow of digital planning and 3D printed custom devices for a poor prognostic freshwater turtle with severe shell damage. At the 12-month follow-up, the favourable outcome suggested that this shell-type device provides protection and precisely fits the defect border with complex geometry. Several clinical reports have demonstrated the treatment of shell injury of freshwater turtles using different appliances. In contrast, there is insufficient information on healing outcomes. Compared with these studies, the present report characterized wound healing conditions by clinical and 3D radiographic findings.

Three-dimensional technology has become feasible and affordable in recent years. The powerful machines become facile and easy to maintain, while the consumable materials provide sustainable strength and easy storage at a reasonable price [13]. This technology has been utilized in many fields of medication with favourable outcomes [14]. This report applied 3D scanning, CAD software, and 3D printing to successfully and rapidly achieve this rescue at a low cost. The 1-year follow-up result further confirms the success of our treatment. These accessible 3D tools with high performance may provide an alternative way to help more animals with limited time and costs and replace human resources. With these 3D tools, the device’s design can evolve quickly along with the need after tests and discussion. The power of CAD software and printer gives us the ability to respond to needs and validate ideas rapidly. When severe shell damage occurs in chelonians, the loss of shell specimens makes wound management more challenging for aquatic and semiaquatic chelonians. Adjunctive with the 3D technique, fabrication of a customized waterproof shield allows for normal husbandry and natural behaviours during shell wound healing.

The shell material and structure are integrated systems that provide load-bearing capacity and protect the internal organs from injury [15]. A previous study analysed the stress distribution of turtle shells using a finite element model [16]. The highest value was located on the inside surface of the four bridges of the turtle shell. The initial 3D imaging showed that the left anterior bridge was fractured (Figure 4A). Therefore, the device’s material should provide strong resistance to the turtle shell. PLA was selected as a leading candidate material owing to its thermoplastic, relatively lightweight, and high-strength mechanical properties [16]. There were still several research limitations. Primarily, this is a single case report, and we did not measure the stress resistance distribution of this device vertically and horizontally. In addition, the longevity of this PLA device was not tested, nor was the application of bacterial cultures for the antibiotic. To make a firm attachment of the hull structure on the defect, we designed snap-in joints [17] in the groove to engage the overhanging shell. Compared with conventional screw fixation, this noninvasive approach avoids anaesthesia complications and the risk of infection. Under the initial condition, the physical condition of the chelonian with severe shell injury was not suitable for anaesthesia, and the bone defect was not stable for using pins or screws. Therefore, we considered some sealants to fill the device frame and the shell seam at the beginning of the device application. PDMS was chosen for this purpose. PDMS is a biocompatible material that has been used in many medical devices [18]. Thanks to its excellent conformability, PDMS is broadly used in the semiconductor industry to replicate surface features down to the subnano scale [19] and is also commercially available as a sealant. With the property of biocompatibility, no adverse effects to aquatic organisms, and resistance to biological fouling [20,21], which is essential when the device is constantly in water, PDMS becomes an adequate sealant to eliminate the risk of infection and chemical toxicity [22]. At a five-month examination, hard tissue healing was generally observed radiographically at the anterior bridge of the defect after application of the device (Figure 4D).

We framed the defect with a hull. The hull design enhanced the framework’s strength to compensate for the weakened shell structure and acted as a watertight armor to protect either the vulnerable coelomic membrane or the clean environment from the contagious but inevitable water immersion. The hull is slightly above the waterline and left with a large opening for subsequent wound management (Figure 2C,D). The opening provided room to reach the wounds inside the defect. The opening with an irregular frame was enclosed by the corresponding cover with a porthole (Figure 2E,F). The cover was designed as a removable part when accessing the fundamental defect.

The porthole on the cover was designed and fabricated with thread that matched a wide bottle cap (Figure 1F) available at local convenience stores. This design followed the previous study, and it is the first case presented in the literature [12]. As a result, the porthole can be neatly opened and closed, and the purulent pocket wound can be easily accessed through the porthole for daily care. In addition, this design avoided frequent disengagement between the cover and hull, which further prevented the failure of the watertight structure. The daily care was manageable with one medical personnel, and the workforce was significantly reduced with this device.

The chelonian shell was still not fully recovered at the 1-year follow-up. However, the CBCT examination indicated that there was a significant improvement in wound healing of hard tissue (Figure 4). The physical condition of the turtle was stable, and it could walk smoothly without restricted movement with the device. The fixation of the device should be observed every month. Continuous maturation and keratinization of the coelomic membrane were observed, and the device was not removed during the 12-month follow-up. In addition, the device could be removed when the shell fracture was fully closed with callus formation and bone healing.

## 5. Conclusions

This report ably demonstrated a rapid and practical process to rescue a severely injured wild freshwater turtle using advanced 3D technology with limited human resources and a relatively low-cost method. Thanks to the maturation of 3D technology, these 3D tools have become robust and affordable. These sophisticated tools gave us the chance to extricate an animal from the risk of euthanasia. This progression delivered an alternative point of view to apply 3D technology to wild animal rescue, and in all of veterinary medicine, which may help us approach new methods from conventional practice.

## Data Availability

Not applicable.
